# Genome-wide Expression Analysis Identifies the Association between SEC14L2 and Castration-resistant Prostate Cancer Survival

**DOI:** 10.7150/jca.50299

**Published:** 2021-02-22

**Authors:** Shiyun Liu, Da Huang, Jingyi Huang, Jiaqi Yan, Tianhe Chen, Ning Zhang, Guangliang Jiang, Yi Gao, Danfeng Xu, Rong Na

**Affiliations:** 1 Department of Urology, Ruijin Hospital, Shanghai Jiao Tong University School of Medicine, Shanghai, China.; 2 Department of Urology, Shanghai General Hospital, Shanghai Jiao Tong University, Shanghai, China.; 3 Program for Personalized Cancer Care, NorthShore University HealthSystem (Teaching affiliation of University of Chicago), Evanston, IL 60201, USA.

**Keywords:** Expression, Castration-resistant prostate cancer, prognosis.

## Abstract

**Background:** Mechanism of castration-resistant prostate cancer (CRPC) is still unclear. Our objective is to investigate the association between genes expression and CRPC through the genome-wide approach and functional researches.

**Methods:** Differentially expressed genes (DEGs) between PCa and CRPC tissues were identified using expression profile obtained from Gene Expression Omnibus database (GEO). Survival analysis was performed using online database Gene Expression Profiling Interactive Analysis (GEPIA). Oncomine database was further used to explore the relationship between DEGs expression levels with clinical parameters. After *in silico* study, SEC14L2-knockdown CRPC cells and normal prostatic epithelial cells were used for *in vitro* study to verify its biological functions.

**Results:** A total of 3 consistently changed DEGs (SEC14L2, DMD, SEL1L) were identified correlating with CRPC after cross validation in three independent datasets. Low expression of SEC14L2 was associated with poorer disease-free survival and higher Gleason score than normal/high expression of SEC14L2. SEC14L2 knockdown promoted cell proliferation, migration, invasion as well as cell cycle progression in CRPC cells (all P<0.05) while no significant effects were observed in normal prostatic epithelial cells.

**Conclusions:** Low expression of SEC14L2 was significantly associated with CRPC, and correlated with PCa aggressiveness and poorer prognosis. SEC14L2 might be a potential biomarker or drug target for CRPC.

## Introduction

Prostate cancer (PCa) has become one of the most common cancers in the world 1. To date, androgen-deprivation therapy (ADT) has remained the most essential and first-line treatment for men with advanced PCa since it was introduced in 1970s 2, 3. Despite its efficacy in treating PCa, most patients ultimately progress to castration-resistant prostate cancer (CRPC) within years. They eventually become insensitive to ADT treatment 4. As a more lethal stage of the disease, CRPC is critically challenging for clinical treatment due to the lack of effective therapies and poor prognosis 4.

Given that the effectiveness of ADT relies on the critical role of androgen receptor (AR) in the progression of PCa, enormous efforts have been devoted to discover other hub genes. So far, scientists have reported that TMPRSS2:ERG fusion gene and deletion of PTEN are associated with poorer prognosis 5, 6. SRD5α2, CYP17, BRAC1 and BRAC2 mutations are found to be related with elevated risk for prostate cancer 5. Splicing variation of AR, overexpression of growth factor receptors and elevated level of YAP, STAT-3 are associated with the development of CRPC 5, 7. Although numerous biomarkers have been reported, most of them are not actionable targets.

In this study, *via* bioinformatic methods based on gene expression databases, we aimed to investigate additional CRPC related molecules and to verify their biological functions in CRPC cell line. Our purpose is to find potential biomarkers and actionable targets in CRPC.

## Materials and Methods

### Microarray Gene Expression Data Analysis

Genome-wide expression microarray data comparing PCa with CRPC were obtained from Gene Expression Omnibus database (GEO) (https://www.ncbi.nlm.nih.gov/geo/). The inclusion criteria includes: (a) accessible microarray data from both PCa and CRPC samples in the dataset; (b) the platform and array used in the study were well accepted, and passed the quality control processes during the experimental period; (c) no treatment effect (either no treatment, or pre-treatment data could be obtained). Finally, expression profiles of CRPC and PCa samples from GSE28403, GSE74367 and GSE101607 were used for further analysis 8-10.

Bioconductor packages in R Software (Version 3.5.0) were used to analyze the array data 11. Differentially expressed genes (DEGs) analyses were performed after normalization based on different platform. Any DEG with P value <0.05 and absolute log2FC≥1 was obtained from each dataset for cross validation among the datasets.

### Validation of DEGs in Clinical Databases

Gene Expression Profiling Interactive Analysis (GEPIA) was used to obtained expression data from The Cancer Genome Atlas (TCGA) and perform survival analysis 12. Oncomine database was used to verify the expression levels of DEGs in PCa among different Gleason scores 13.

### Functional Experiments

#### Cell line and culture

DU145 (castration-resistant prostate cancer cell), PC3 (castration-resistant prostate cancer cell) and RWPE-1 (normal prostatic epithelial cell) were purchased from the Cell Bank of the Shanghai Institute of Biochemistry and Cell Biology, Chinese Academy of Sciences (Shanghai, China). Cells were cultured as protocol in RPMI-1640 medium (Gibco, Grand Island, NY, USA) with 10% FBS at 37℃ in the environment with 5% CO2. RWPE-1 cells were grown in Keratinocyte Serum Free Medium (Gibco, Grand Island, NY, USA) at 37℃ in the environment with 5% CO2.

#### Lentivirus vectors for SEC14L2 small hairpin RNA (shRNA) construction and transfection

Three SEC14L2 shRNA lentivirus vectors and the control vector with green fluorescent protein (GFP) were purchased from GeneChem Co.,Ltd (Shanghai, China). DU145, PC3 and RWPE-1 cells in the logarithmic growth phase were harvested and seeded in 6-well plates. After cells grew at about 20% confluence, lentiviruses were used for transfection according to the manufacturer's protocols. Fluorescence images were taken 72h after the transfection and RT-qPCR was used to validate the interference efficiency.

#### RT-qPCR

Trizol agent (Pufei, Shanghai, China) was used to extract total RNA from DU145, PC3 and RWPE-1 cells and mRNA reverse transcription was then implemented using M-MLV Reverse Transcriptase kit (Progema, Madison, USA). RNA quality and quantification were assessed spectrophotometrically with a 260/280 ratio of >1.8. Primers for SEC14L2 and GAPDH were synthesized by GeneChem Co.,Ltd (Shanghai, China) and sequences were shown as follow: SEC14L2 sense 5'-CGTCAATGTTGGCTACTCT-3', antisense 5'-ACAATCCTGGGTTCAAATC -3', GAPDH 5'- TGACTTCAACAGCGACACCCA -3', antisense 5'-CACCCTGTTGCTGTAGCCAAA-3'. RT-qPCR using SYBR Master Mixture (TAKARA, Dalian, China) was performed to detect mRNA expression. The reaction system (12μl) contained 6μl SYBR premix ex taq, 0.5μl forward primer, 0.5μl reverse primer, 1μl DNA template and 4μl RNase-Free water. The relative mRNA level of SEC14L2 were standardized to GAPDH based on 2^-ΔΔCt^ method.

#### CCK-8 assay

DU145, PC3 and RWPE-1 cells were seeded in 96-well plates at a density of about 2500 cells/well and the supernatant was replaced with complete medium containing 10% CCK-8 agent 2h before detection. After incubation at 37℃ for 2h, the absorbance was measured by microplate reader at 450nm. The proliferation rates were tested at 1, 2, 3, 4d after transfection.

#### Clone formation assay

DU145, PC3 and RWPE-1 cells transfected with si-SEC14L2 or control vector were seeded in 6-well plates at a concentration of 500 cells/well. Clones were fixed with paraformaldehyde and stained with crystal violet after 14 days.

#### Flowcytometry assay

Cells were harvested when reaching about 80% confluency and washed twice with ice-cold PBS. Cells were then incubated with 1000μl PBS containing 25μl propidium iodide (2mg/ml, Sigma-Aldrich), 10μl RNase A (100μg /ml, Thermo Fisher) and 40μl Triton X-100 (Sigma-Aldrich) for 30 minutes at room temperature and analyzed with BD Accuri C6 plus flow cytometer (BD Biosciences).

#### Transwell migration and invasion assay

For the migration assays, the 24-well Transwell chamber (Corning, USA) was used and 100μl complete medium containing 1
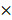
10^5^ cells were added to the upper chambers while 600μl RPMI-1640 medium with 30% FBS was added to the lower chambers. After culture for 24 hours, cells remaining at the inner face of the chamber were removed and those on the outer membrane were fixed with 4% paraformaldehyde, labelled with crystal violet and counted with inverted fluorescence microscope.

For the invasion assays, the sample procedure was performed except the inner face of the upper chamber was coated by Matrigel.

### Statistical analysis

R software (Version 3.5.0) were used to perform DEGs analyses. Heatmap was used to describe the differential expression level. Cross validation among the datasets were performed and presented using vennDiagram analysis. SPSS 19.0.0 software (IBM SPSS, USA) was used to analyze all the experimental data. In the functional study, two-tail Student's T test was implemented and P-value<0.05 was considered as statistical significance. All experiments were triplicated for validation.

## Results

Three GEO datasets (GSE28403, GSE74367, GSE101607) were investigated to explore differential expressed mRNAs between CRPC and PCa samples. A total of 94 CRPC (45 in GSE74367, 40 in GSE101607 and 9 in GSE28403 using Affymetrix Genechip platform) and 23 PCa samples (11 in GSE74367, 8 in GSE101607 and 4 in GSE28403 using Affymetrix Genechip platform) were obtained for further analysis. Details of the datasets were shown in supplementary Table 1.

**Figure 1:**
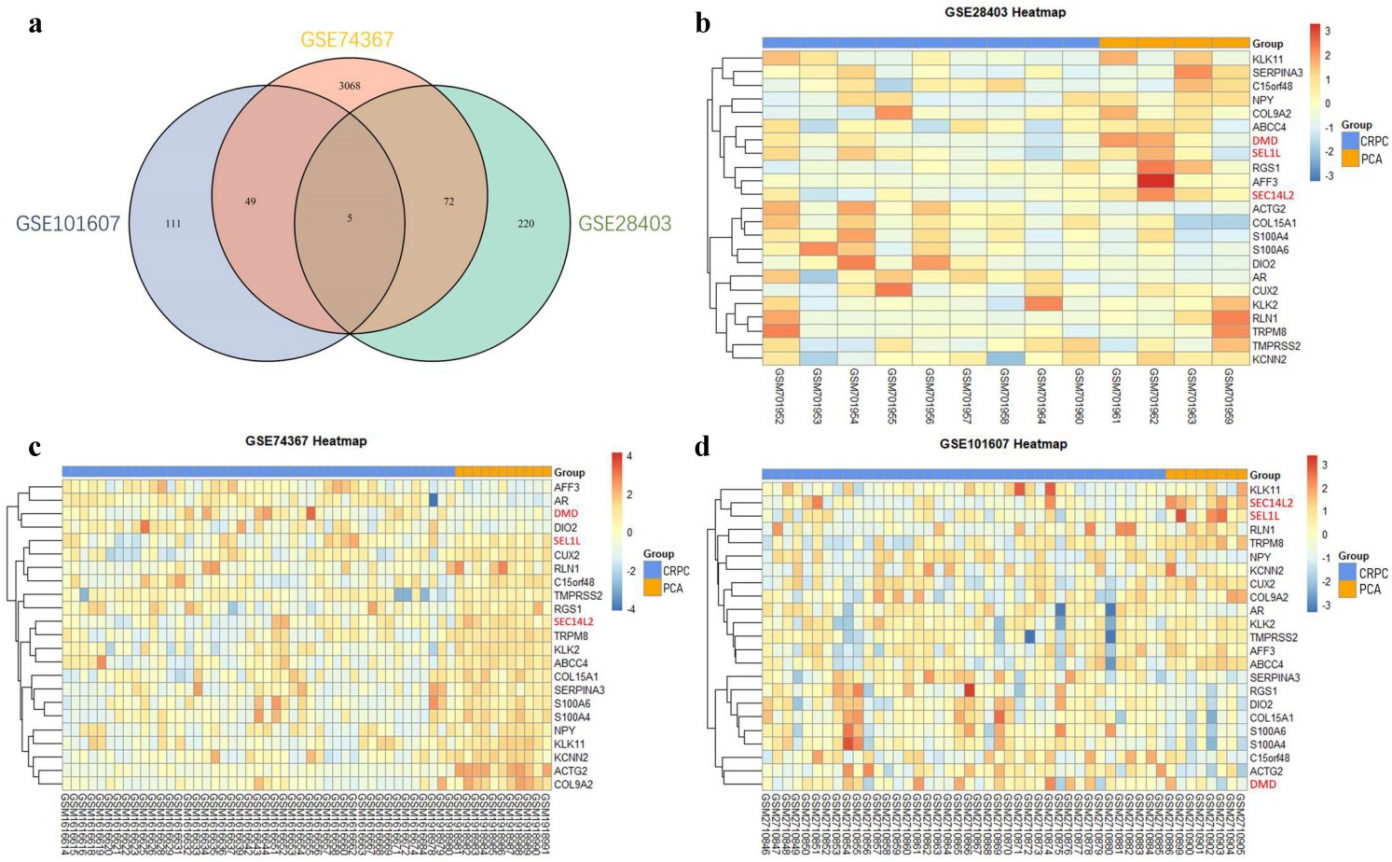
Identification of differentially expressed genes. (a) Venn plot showing 5 common DEGs among GSE28403, GSE74367 and GSE101607. (b, c and d) Volcano plot showing DEGs in CRPC compared with PCa samples. The gradual color ranging from blue to red represents the process from downregulation to upregulation. The consistently changed 3 DEGs (DMD, SEC14L2 and SEL1L) were highlighted in red.

After the normalization, 297 DEGs were identified in GSE28403, 3194 DEGs were identified in GSE74367 and 165 DEGs were identified in GSE101607, using the criteria of P value <0.05 and absolute log2FC ≥1. A total of 5 shared DEGs were further sorted using Venndiagram, of which DMD, SEC14L2 and SEL1L were consistently downregulated (Fig. 1a and Supplementary Table 2). The heatmap plot was used to present DEGs respectively (Fig. 1b, c and d).

**Figure 2:**
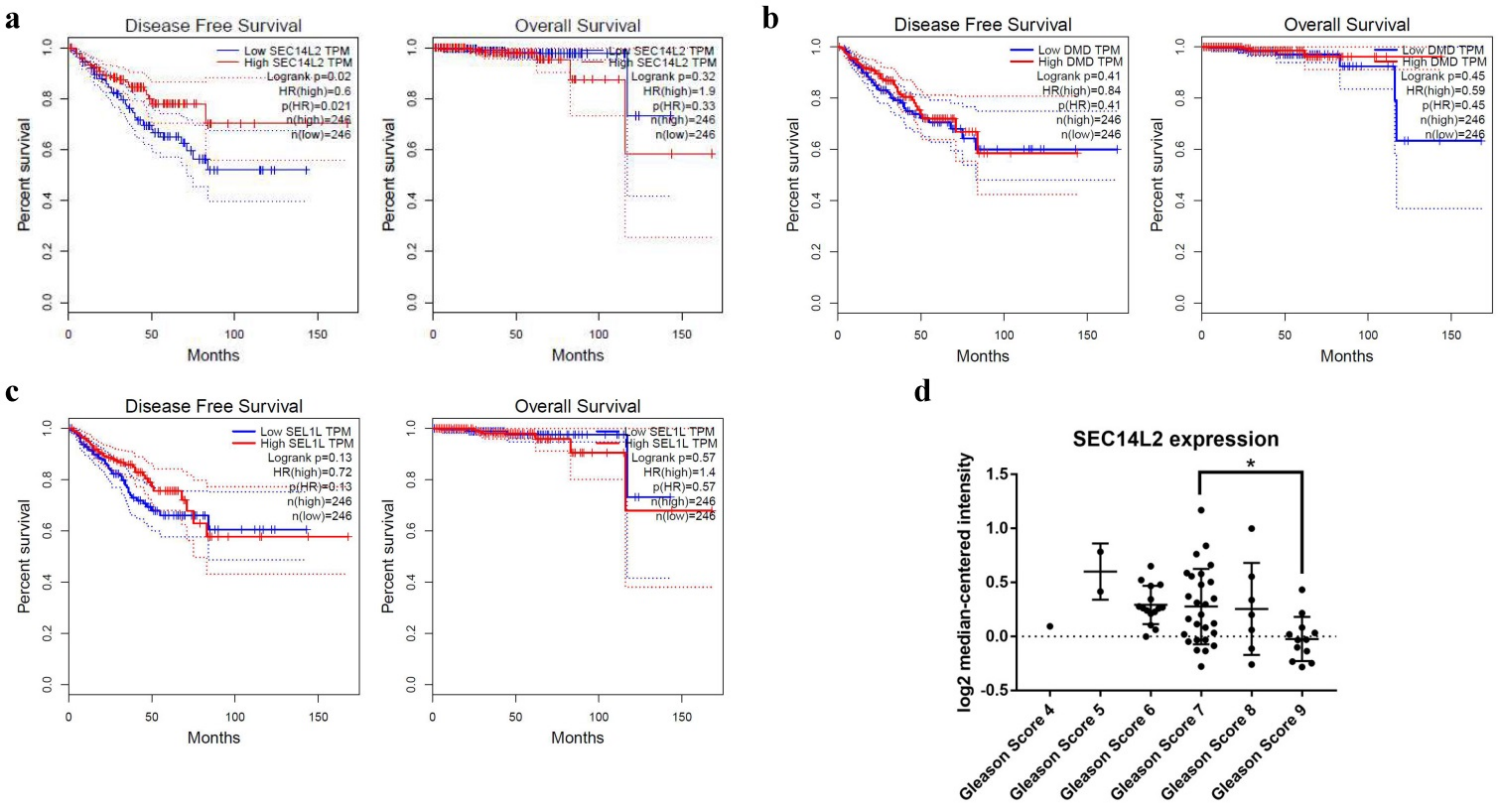
The correlation between DEGs expression and clinical parameters. (a) SEC14L2 downregulation was associated with shorter DFS survival but had no effect on OS. (b and c) Neither DMD nor SEL1L had siginificant effect on PCa survival. (d) The Oncomine dataset demonstrated SEC14L2 expression declined with the increase of Gleason score. SEC14L2 expression in Gleason score 9 samples was siginificantly lower than Gleason score 7. *p<0.05 were considered as statistically significant.

To further explore the relationship between co-differentially expressed DEGs with the prognosis of PCa, GEPIA database was used to perform survival analysis. The results showed that high expression of SEC14L2 was associated with longer disease free survival of PCa (HR=0.6, p<0.05, Fig. 2a), while the other two genes had no significant association with disease prognosis (p>0.05, Fig. 2b, c). Additionally, Oncomine database was further applied to reveal the association between SEC14L2 expression and clinicopathological features (Fig. 2d). Consistent with survival analysis, the Gleason score upgraded along with the decline of SEC14L2 expression (Gleason score 7 vs. Gleason score 9, p<0.05) 14.

**Figure 3:**
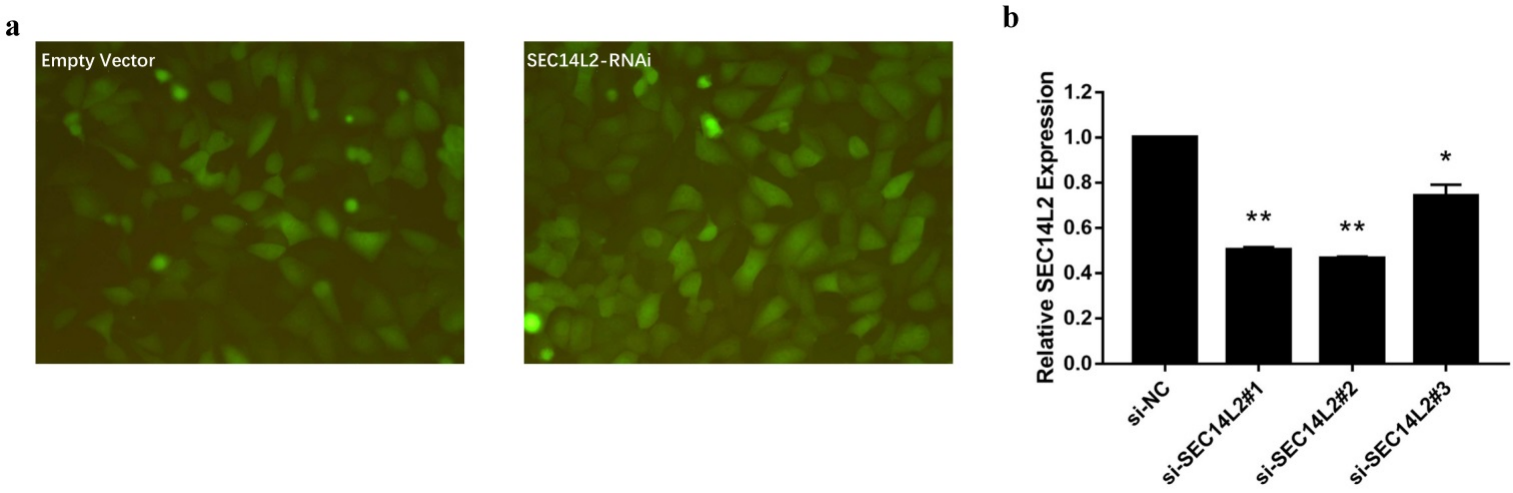
Verification of SEC14L2 knockdown in DU145 cells. (a) Fluorescence microscope images of DU145 cells 72h after transfection with or without SEC14L2 RNAi. (b) Relative SEC14L2 mRNA expression level normalized to GAPDH in different shRNA groups or empty vector. * p<0.05, ** p<0.01 were considered as statistically significant.

We then knocked down (KD) SEC14L2 by introducing shRNAs targeted against SEC14L2 into DU145 (a CRPC cell line) via lentivirus vector system. GFP was set as a reporter gene. Fluorescence microscope images of the exposed DU145 cells were used to evaluate the transfection efficiency. The results showed that more than 80% cells expressed GFP, indicating a high transfection efficiency of lentiviral system (Fig. 3a). RT-qPCR was further implemented to verify the silencing effect of shRNAs and the results confirmed that SEC14L2 was successfully downregulated (p<0.05). In addition, si-SEC14L2#1 and si-SEC14L2#2 showed similar interference effect but both were more potent than si-SEC14L2#3. Therefore, si-SEC14L2#2 was chosen for further experiments (Fig. 3b). The shRNA was then introduced to PC3 and RWPE-1 cells and decreased expressions of SEC14L2 were verified by RT-qPCR (Fig. S2).

**Figure 4:**
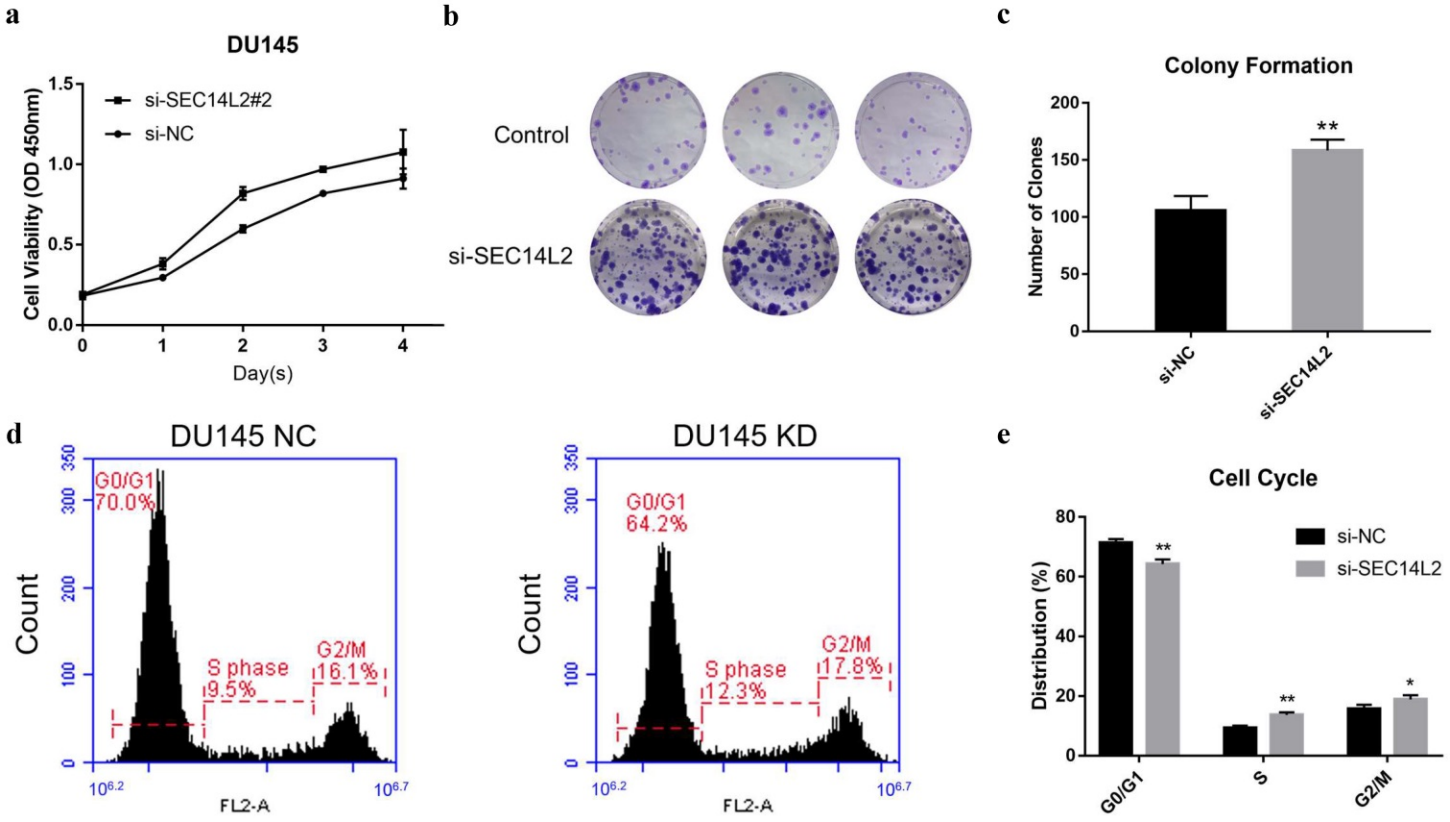
SEC14L2 knockdown promoted DU145 cell proliferation. (a) CCK8 assays revealed that downregulation of SEC14L2 promoted the growth rate of DU145. (b, c) Downregulation of SEC14L2 increased the number of clones in DU145 cells. (d, e) Downregulation of SEC14L2 fueled the G1-to-S phase transition. *p<0.05, **p<0.01 were considered as statistically significant.

CCK8 assay and clone formation assay was performed to evaluate the impact of SEC14L2 expression on the proliferation capacity of DU145 cells. The results of CCK8 assay revealed that the cell growth rate in SCE14L2 KD group was significantly promoted comparing with control group (p<0.05, Fig. 4a). Similarly, more clones were observed in the SEC14L2 KD group (NC vs KD 105±7 vs 158±5, p<0.05, Fig. 4b, c). Downregulation of SEC14L2 also promoted clonogenic growth of PC3 cells (NC vs KD 121±5 vs 153±6, p<0.05, Fig. S3e, f) but not RWPE-1 cells (NC vs KD 74±2 vs 83±3, p<0.05, Fig. S3b, c). Flow cytometry was then utilized for cell cycle analysis. We observed a larger proportion of SEC14L2 KD cells in the S and G2 phase, as known as a mitotic active stage. Meanwhile, more cells in in the G1 phase were found in the control group (p<0.05, Fig. 4d, e). The similar G1-to-S transition was also identified in PC3 cells (p<0.05, Fig. S4c, d). However, SEC14L2 downregulation had no significant effect on the cell cycle of RWPE-1 cells (Fig. S4a, b). These results suggested that SEC14L2 KD may enhance mitosis in CRPC cells.

**Figure 5:**
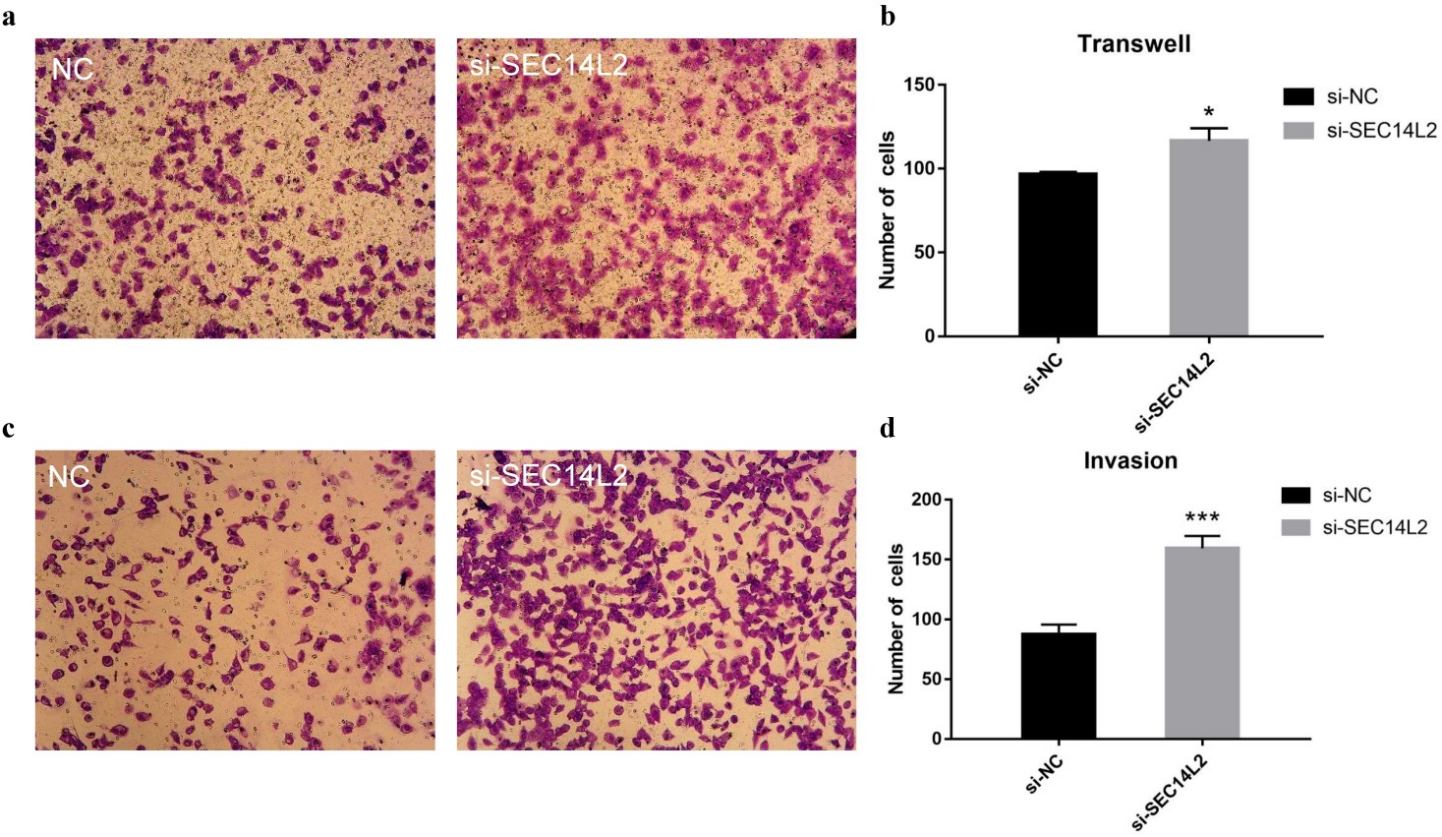
SEC14L2 knockdown promoted DU145 mobility. (a) Transwell migration images of SEC14L2 silenced group and control group (b) SEC14L2 KD enhanced cell mobility (c) Transwell invasion images of SEC14L2 silenced group and control group (d) SEC14L2 KD enhanced cell invasion capacity. *p<0.05, ***p<0.001 were considered as statistically significant.

To further investigate the role of SEC14L2 in cancer progression, cell migration assays and cell invasion assays were performed. Compared with control group, more DU145 cells were observed on the lower chamber after 24 hours in the SEC14L2 KD group (NC vs KD 96±1.56 vs 117±7.56, p<0.05), indicating a strengthened motor capacity (Fig. 5a, b). The similar phenomenon was also observed in PC3 cells (NC vs KD 94±2.03 vs 121±5.78, p<0.05, Fig. S5a, b). Afterwards, the upper chambers were covered by Matrigel so as to mimic the basement membrane and the results confirmed the enhanced invasion capacity of SEC14L2 downregulated DU145 cells (NC vs KD 88±7.93 vs 159±10.47, p<0.05, Fig. 5c, d). Likewise, more PC3 cells in the SEC14L2 KD group invaded through Matrigel (NC vs KD 80±3.18 vs 141±6.74, p<0.05, Fig. S5c, d). Above phenomena proved that interference of SEC14L2 expression markedly promote CRPC cells migration and invasion.

## Discussion

After a period of ADT treatment, disease will always develop to a more malignant and lethal stage, as known as CRPC 4. With the high throughput sequencing and bioinformatics advancement being widely applied, researchers are able to investigate additional biomarkers or actionable targets via open access databases. In the present study, we identified 3 DEGs (DMD, SEC14L2 and SEL1L) that are associated with CRPC by analyzing three independent series from GEO database. Cross validation *via* venn diagram, survival analysis and clinicopathological correlation study further confirmed that only SEC14L2 was associated with CRPC aggressiveness and survival. Further functional experiments proved that SEC14L2 knockdown promoted cell proliferation, migration, invasion as well as cell cycle progression in CRPC cells but not in normal prostatic epithelial cells. Taken together, these results implicated the potential tumor suppressive role of SEC14L2 in CRPC.

As one of the six members of SEC14 family, SEC14L2 encodes lipid binding proteins such as α-tocopherol transfer protein, which facilitates the uptake of Vitamin E 15. Though SEC14L2 expression is relatively low in many human tissues, it is highly expressed in liver, brain, small intestine and prostate 16. Previous studies demonstrated that low expression of TAP (protein encoded by SEC14L2) was associated with higher Ki-67 expression in prostate cancer tissue. Lower expression of TAP was also found to be related with elevated PSA level, larger tumor size, higher clinical stage and poorer survival 17. Our study further confirmed these findings. In additional, we found that low expression of SEC14L2 was significantly associated with disease aggressiveness and poorer survival.

Vitamin E, which acts as the substrate of TAP has been reported as a protective factor in prostate cancer, especially in smokers or those in advanced stage 18-22. Whereas, other studies proclaim that Vitamin E supplement at a dosage of 400 IU/day may not protect males from prostate cancer 23, 24 or even increase the prostate cancer risk 25. Some researchers believe that limited understanding of metabolic and physiologic mechanism behind Vitamin E shall account for the controversial role of Vitamin E in the prostate cancer 26, 27. *In vitro* experiments confirm that SEC14L2 suppresses PCa cells growth by facilitating the uptake of Vitamin E and α-Tocopheryl succinate, an analogue of Vitamin E with proapoptotic effect 28, 29. Therefore, given the inhibitory effect SEC14L2 posed on CRPC cell lines and the α-tocopherol transfer protein it encoded, we could safely speculate that SEC14L2 coupled with Vitamin E or its analogues might be conducive to the development of next generation therapy. Vitamin E may also be a supplement for localized PCa patients which will protect them from developing CRPC. Further study based on animal model and population level should be conducted for this hypothesis.

Some limitations should be noted in our study. The sample size was relatively small. However, based on the 3 independent studies, we were able to find 3 differentially expressed genes (DMD, SEC14L2 and SEL1L) that were significantly associated with CRPC in each of the dataset. In the further investigation, we would also like to confirmed our findings via *in vivo* experiments and population-based studies.

## Conclusion

Low expression of SEC14L2 was significantly associated with CRPC, and correlated with PCa aggressiveness and poorer prognosis. SEC14L2 might be a potential biomarker or drug target for CRPC.

## Supplementary Material

Supplementary figures and tables.
