# Supplementary material for: Genome-wide Expression Analysis Identifies the Association between SEC14L2 and Castration-resistant Prostate Cancer Survival
Source: J Cancer. 2021 Feb 22;12(8):2173–80. doi: 10.7150/jca.50299 (PMC7974876; doi:10.7150/jca.50299)
Supplement: Supplementary file 1 — Supplementary figures and tables. [file jcav12p2173s1.pdf]

Supplementary Table 1

Details about three microarray datasets used in this study

Study	Year	Platform	Sample No.		
			Total	PCa	CRPC
GSE28403	2011	GPL570	13	4	9
GSE74367	2016	GPL15659	56	11	45
GSE101607	2017	GPL10558	48	8	40

Supplementary Table 2

The intersection of three datasets

Gene Symbol	Full Name
DMD	Dystrophin
SEC14L2	SEC14 like lipid binding 2
SEL1L	SEL1L adaptor subunit of ERAD E3 ubiquitin ligase

Supplementary Figure 1

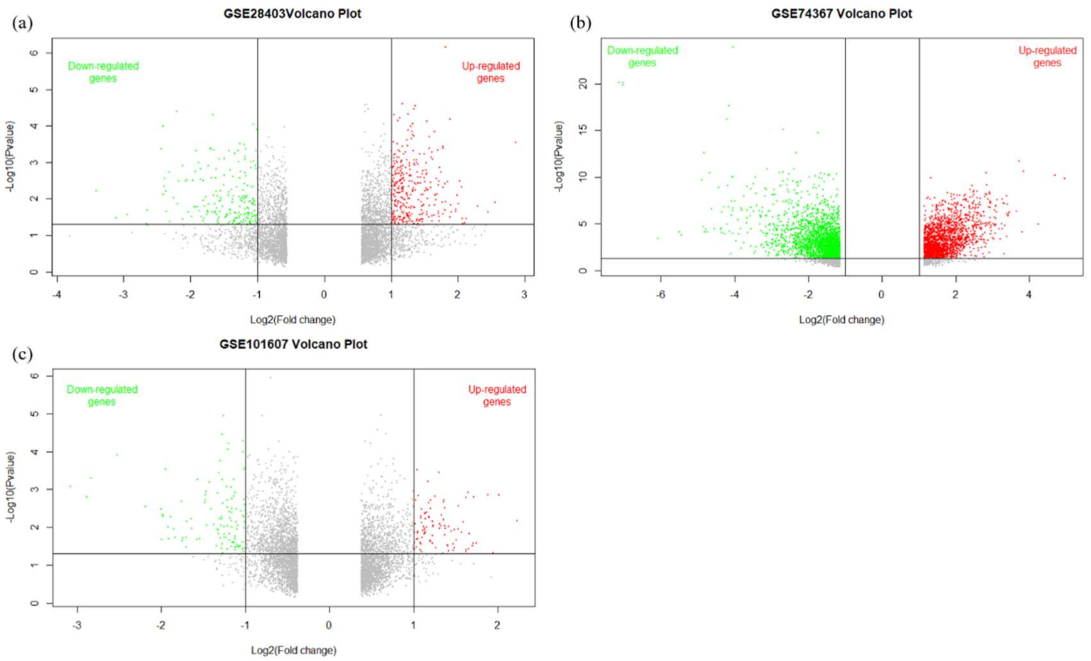

Volcano plots showing all the differentially expressed genes (a) GSE28403; (b) GSE74367; (c) GSE101607. Grey represents non-significantly changed genes, green represents down-regulated ones and red represents up-regulated ones according to the threshold of $P < 0.05$, $|logFC| > 1$.

Supplementary Figure 2

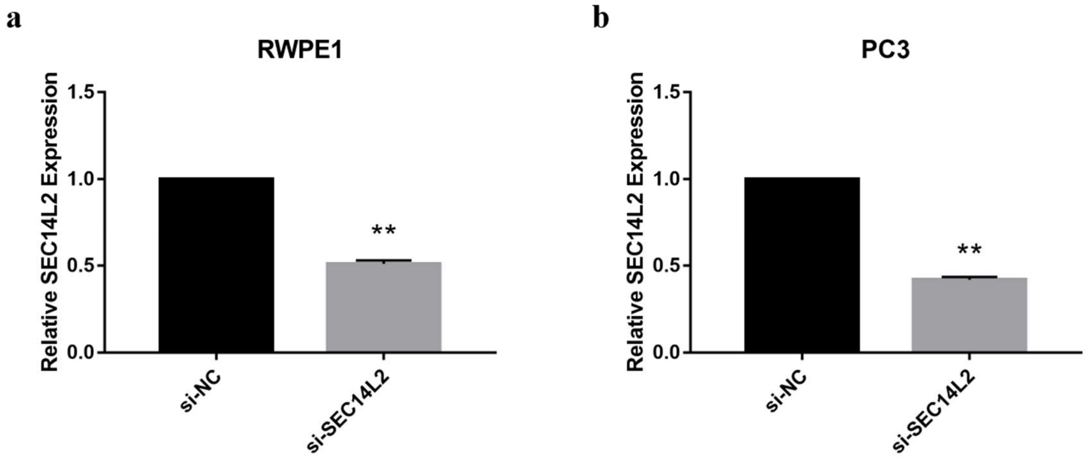

Verification of SEC14L2 knockdown in RWPE1 and PC3 cells. (a) Relative SEC14L2 mRNA expression level normalized to GAPDH in empty vector or shRNA group in RWPE1 cells. (b) Relative SEC14L2 mRNA expression level normalized to GAPDH in empty vector or shRNA group in PC3 cells. **p<0.01 was considered as statistically significant.

Supplementary Figure 3

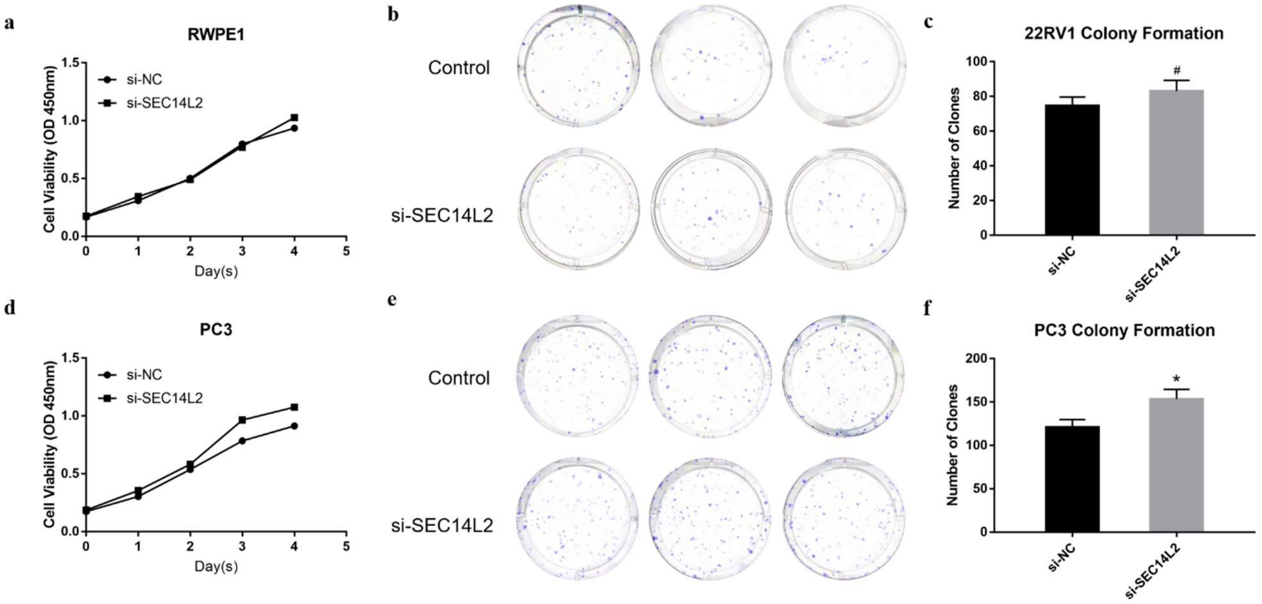

SEC14L2 downregulation promoted CRPC cells growth. (a) SEC14L2 KD had no significant effect on the proliferation of RWPE1 cells. (b, c) SEC14L2 KD didn’t boost colony formation capacity of RWPE1 cells. (d) SEC14L2 KD promoted PC3 cells proliferation. (e, f) SEC14L2 KD increased the number of clones in PC3 cells. #p>0.05 was considered as not statistically significant while *p<0.05 was considered as statistically significant.

Supplementary Figure 4

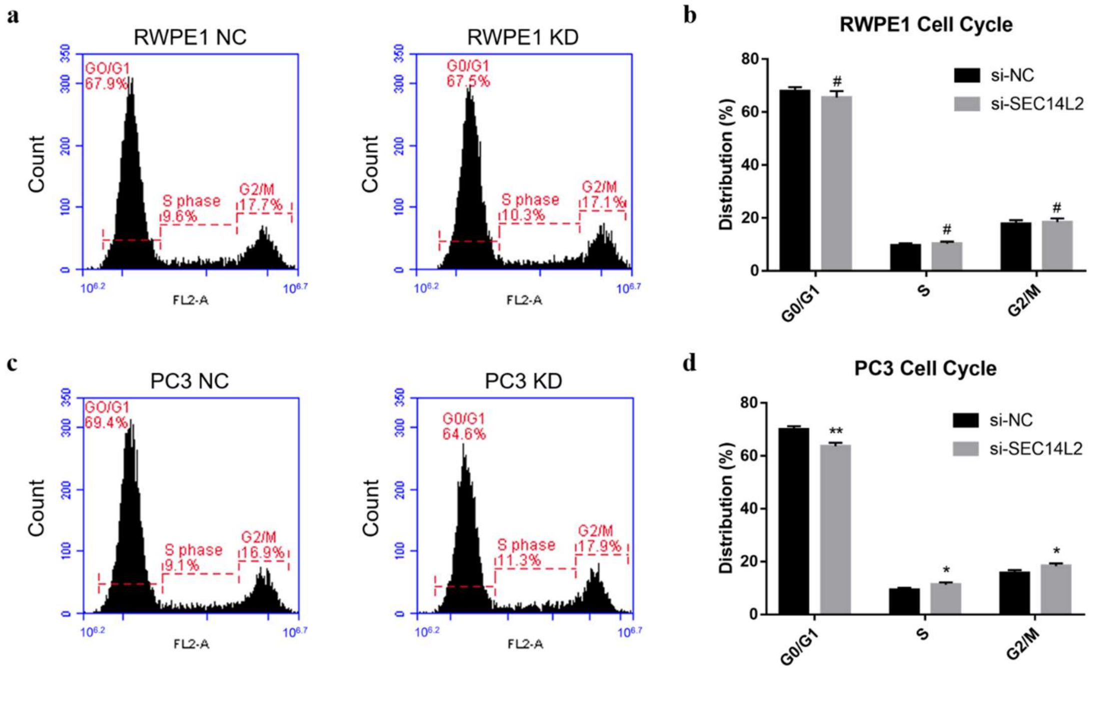

SEC14L2 downregulation promoted G1-to-S transition in CRPC cells. (a, b) SEC14L2 KD had no significant effect on the cell cycle of normal prostatic epithelial cells. (c, d) SEC14L2 downregulation promoted PC3 cells G1-to-S transition. #p>0.05 was considered as not statistically significant while *p<0.05 was considered as statistically significant.

Supplementary Figure 5

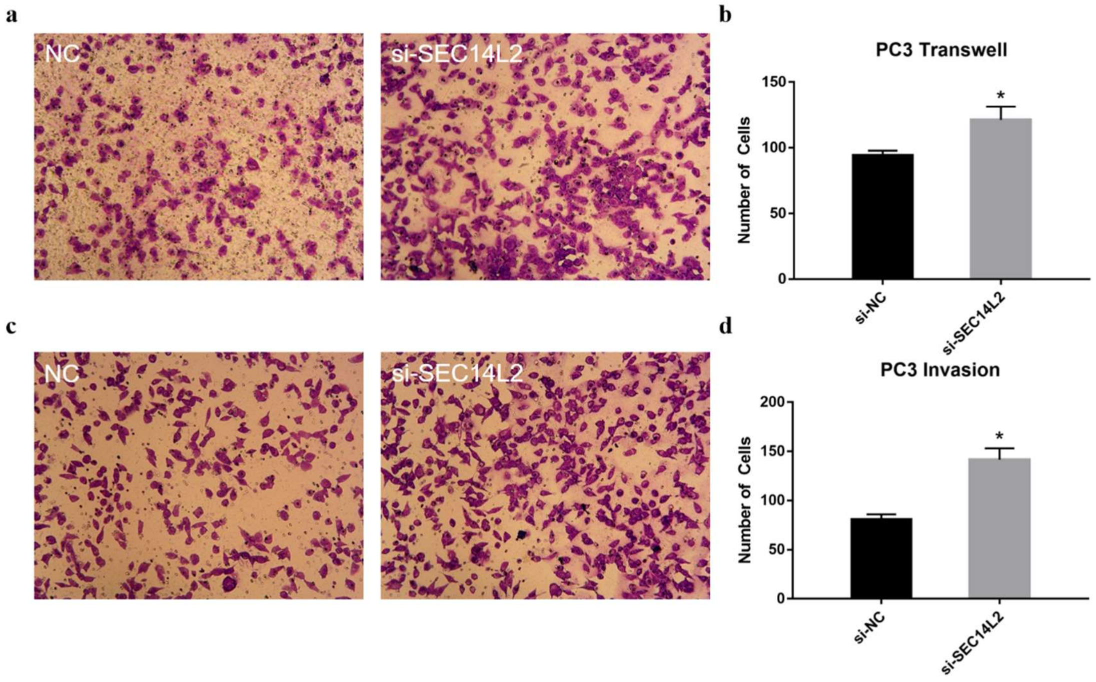

SEC14L2 knockdown promoted PC3 mobility. (a) Migration images of PC3 cells in NC group and KD group. (b) SEC14L2 KD enhanced PC3 cells mobility. (c) Invasion images of PC3 cells in

NC group and KD group. (d) SEC14L2 KD enhanced PC3 cells invasion. *$p<0.05$ was considered as statistically significant.
